# Expression of Vitamin D Receptor (VDR) Positively Correlates with Survival of Urothelial Bladder Cancer Patients

**DOI:** 10.3390/ijms161024369

**Published:** 2015-10-15

**Authors:** Wojciech Jóźwicki, Anna A. Brożyna, Jerzy Siekiera, Andrzej T. Slominski

**Affiliations:** 1 Department of Tumour Pathology and Pathomorphology, Nicolaus Copernicus University Collegium Medicum in Bydgoszcz, Bydgoszcz 85-796, Poland; E-Mail: anna.brozyna@cm.umk.pl; 2 Department of Tumour Pathology and Pathomorphology, Oncology Centre–Prof. Franciszek Łukaszczyk Memorial Hospital, Bydgoszcz 85-796, Poland; 3 Department of Urology, Oncology Centre–Prof. Franciszek Łukaszczyk Memorial Hospital, Bydgoszcz 85-796, Poland; E-Mail: siekieraj@co.bydgoszcz.pl; 4 Departments of Dermatology and Pathology, University of Alabama at Birmingham, Birmingham, AL 35294, USA; E-Mail: aslominski@uabmc.edu; 5 Department of Veteran Affairs (VA) Medical Center, Birmingham, AL 35294, USA

**Keywords:** vitamin D receptor (VDR), urothelial bladder cancer, survival, vitamin D, CYP27B1

## Abstract

Vitamin D3 shows tumoristatic and anticancer effects by acting through the vitamin D receptor (VDR), while hydroxylation of 25-hydroxyvitamin D3 at position 1α by CYP27B1 is an essential step in its activation. The expression of both the VDR and CYP27B1 has been found in many normal and cancer tissues, but there is a lack of information about its expression in human bladder cancers. The aim of the present research was to examine whether the expression of the VDR and CYP27B1 in bladder cancer was related to the prognostic markers and disease outcome. We analyzed VDR and CYP27B1 in samples of tumor and normal tissues obtained from 71 urinary bladder cancer patients. The highest VDR immunostaining was found in normal epithelium and was significantly lower in bladder cancer cells (*p* < 0.001 with Mann–Whitney *U* test). VDR expression was lowest in more advanced (pT2b–pT4) (*p* = 0.005 with Mann–Whitney *U* test) and metastasizing cancers (*p* < 0.05 and *p* = 0.004 with Mann–Whitney *U* test for nuclear and cytoplasmic VDR immunostaining, respectively). The lack of cytoplasmic and nuclear VDR was also related to shorter overall survival (for cytoplasmic VDR immunolocalization 13.3 *vs.* 55.3 months of survival, HR = 1.92, *p* = 0.04 and for nuclear VDR immunostaining 13.5 *vs.* 55.3 months of survival, HR = 2.47, *p* = 0.002 with Mantel-Cox test). In cases with the lack of high cytoplasmic VDR staining the non-classic differentiations (NDs) was observed in higher percentage of tumor area. CYP27B1 expression was lower in cancer cells than in normal epithelial cells (*p* = 0.03 with Mann–Whitney *U* test), but its expression did not correlate with tumor stage (pT), metastasizing, grade, mitotic activity or overall survival. In conclusion, expression of the VDR and CYP27B1 are deregulated in urothelial bladder cancers. Although our results showing a relationship between the decreased VDR expression and prognostic markers and survival time indicate potential usefulness of VDR as a new indicator of a poorer prognosis, further studies are needed in different patient cohorts by independent groups to validate this hypothesis. We also suggest that vitamin D-based therapies may represent an adjuvant strategy in treatment for bladder cancers expressing VDR.

## 1. Introduction

Urothelial bladder cancers are tumors with different biological behavior that depends on the depth of invasion [1]. Papillary tumors confined to the urothelium and tumors confined to the mucosa tend to exhibit a weak invasive tendency, whereas cancers infiltrating the deeper layers of the bladder wall (muscle-invasive bladder cancer; MIBC) show a high metastatic potential [1,2]. MIBC is the fourth most common solid tumor type in men [3]. The basic treatment for MIBC is cystectomy and chemotherapy, and treatment modalities for these patients have not changed significantly in the past few decades. Radical cystectomy is an efficient treatment only for patients with localized MIBC who are lymph node negative, resulting on average in a 70% five-year disease-free survival [4]. In patients with more advanced primary tumors (>pT2) the DFS is shorter (about 50%) after radical cystectomy [4,5]. Chemotherapy may be an optional adjuvant treatment, however, 40%–50% of MIBC patients are not eligible for cisplatin-based combination chemotherapy, because of renal dysfunction [5,6]. Therefore, new, safer treatment modalities that would be suitable for a wider group of bladder cancer patients are needed.

Vitamin D undergoes sequential hydroxylations at C25 (by CYP2R1 and/or CYP27A1) and subsequent final hydroxylation at C1α mediated by CYP27B1 that produces its biologically active form 1,25-dihydroxyvitamin D3 (1,25(OH)_2_D_3_) [7]. 1,25(OH)_2_D_3_ plays a crucial role in the regulation of calcium and phosphate homeostasis and bone mineralization [7,8,9]. In addition, vitamin D affects a broad range of cellular processes involved in the regulation of a variety of cellular functions including cell differentiation, DNA repair, apoptosis, metabolism, oxidative stress, and systemic body homeostasis [7,8,9]. Decreased vitamin D level, expression of the vitamin D receptor (VDR), and deregulation of the function of vitamin D-metabolizing enzymes are all related to serious diseases such as autoimmune diseases, neurologic disorders including schizophrenia, type 2 diabetes mellitus, increased mortality, multiple sclerosis, and others (reviewed in [8,9,10,11,12,13,14,15]). There is a growing body of evidence that the biology of human cancers is affected by vitamin D body endocrine status (reviewed in [8,14]). Similarly, disturbances of the expression of the VDR and enzymes regulating vitamin D activation (CYP27B1) and metabolism (CYP24A1) are linked to the aggressiveness and outcomes of some malignant tumors [16,17,18,19,20]. It was recently reported that vitamin D insufficiency is linked to a higher risk of cancer, including bladder cancer [21,22,23,24], and that the highest risk was found in smokers [22,24]. Several experimental studies have shown that vitamin D and its derivatives have antitumorigenic potential [22,25,26,27,28]. Similarly, in cancer patients, vitamin D supplementation might improve cancer treatment and ameliorate cancer related side effects [29,30,31].

There is a shortage of information about the expression of the VDR expression in relation to the clinical and pathomorphological features. Therefore, the aim of this study was to evaluate the relationship between expression of the VDR and CYP27B1 and prognostic markers (such as tumor advancement, presence of metastases, tumor grade, mitotic activity, non-classic differentiation) in urothelial bladder cancer and its clinical outcome.

## 2. Results

### 2.1. VDR and Bladder Cancer

**Figure 1:**
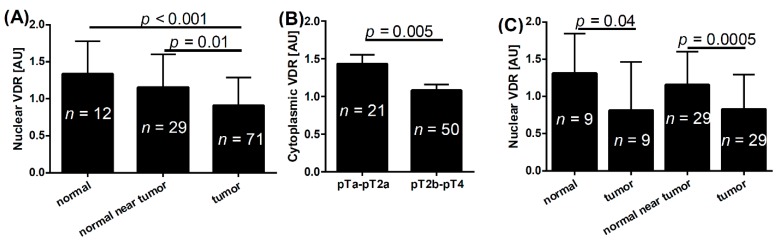

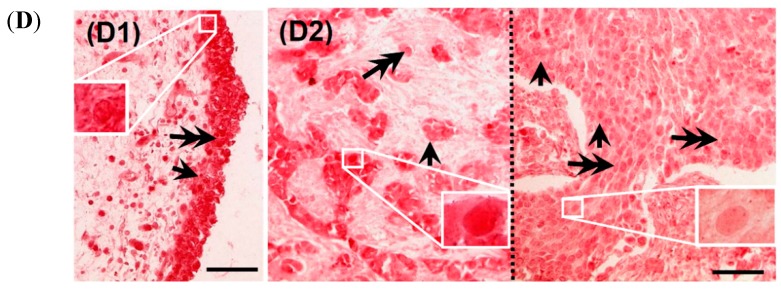
(**A**) Mean nuclear vitamin D receptor level in normal epithelium, normal epithelium surrounding tumors and tumors; (**B**) mean cytoplasmic VDR level in pTa–pT2a and pT2b–pT4 urothelial bladder cancers; (**C**) matched-pair comparison of nuclear VDR in normal epithelium, normal epithelium near tumor and tumor cells; (**D**) representative VDR staining in normal epithelium (**D1**); and cancers (**D2**). Double arrows indicate nuclear VDR immunostaining, arrows indicate cytoplasmic VDR immunostaining, the dotted line separates photos from two different cancers (pT2a (**left**) and pT3a (**right**)). Inserts represent enlarged cells indicated by white squares. AU, arbitrary units; Scale bar: 100 μm; *n*, number of patients.

The VDR was consistently expressed in normal epithelial cells with variable expression in urothelial bladder cancer cells. In all normal samples analyzed, the VDR was localized to the cell nuclei and/or cytoplasm. In malignant cells VDR was also found in cell nuclei and/or cytoplasm. However, a combined lack of nuclear and cytoplasmic VDR was seen in nine of 71 cancer patients (12.7%). The nuclear VDR was absent in nine (12.7%), and the cytoplasmic VDR was absent in 22 cases (31%). The VDR immunostaining in tumor samples differed from normal tissues only for nuclear staining with the highest VDR expression observed in normal epithelial cells (Figure 1A,D1,D2). Matched-pair analysis for nuclear localization of VDR also showed statistically higher expression in normal epithelial cells located 2 cm from the tumor (Wilcoxon matched-pairs signed rank test *p* = 0.04) and in normal epithelium located near (0.5–2.0 cm) the tumor (Wilcoxon matched-pairs signed rank test *p* = 0.0005) (Figure 1C).

VDR expression was also analyzed in relation to the pathomorphological advancement and we found that the cytoplasmic VDR levels were higher in pTa–T2a tumors compared with samples from more advanced cancers (pT2b–pT4) (Figure 1B).

**Figure 2:**
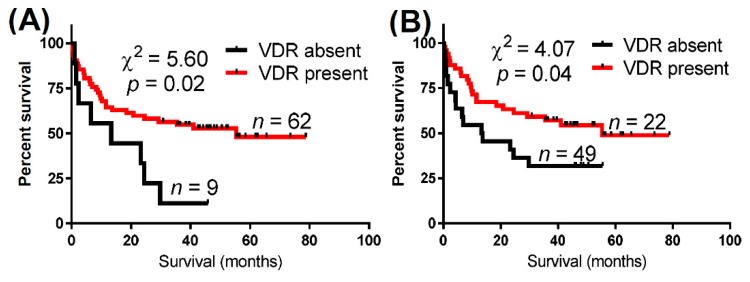
Comparison of overall survival (OS) curves of urothelial bladder patients in relation to the expression of the vitamin D receptor (VDR) in cancer cell nuclei (**A**) and cytoplasm (**B**). *n*, number of patients.

Next, we analyzed the VDR levels in relation to overall survival (OS) of urothelial bladder cancer patients (Table 1, Figure 2). The presence of the VDR in either nuclei or cytoplasm correlated with a significantly longer OS (Table 1, Figure 2). These differences were stronger for nuclear VDR (Figure 2A, Table 1) in comparison to cytoplasmic VDR expression (χ^2^ = 5.6, *p* = 0.02 *vs.* χ^2^ = 4.07, *p* = 0.04) (Figure 2B, Table 1). The detailed results of the overall survival analysis are presented in Table 1. Longer overall survival was observed in both pTa–pT4 patients (Table 1) and those with invasive tumors only (pT1–4), but for the latter the statistical differences were found only for cytoplasmic VDR (χ^2^ = 4.9, *p* = 0.03 by Mantel–Cox test and (χ^2^ = 6.0, *p* = 0.01 by Gehan–Breslow–Wilcoxon Test, mean survival time 5.6 *vs.* 55.3 months for cases without and with cytoplasmic VDR).

**Table 1:** Comparison of overall survival (OS) time in pTa-pT4 urothelial bladder cancer patients in relation to vitamin D receptor (VDR) expression (log-rank Mantel–Cox test).

VDR Expression		Total Cases (*n*)	Deaths (*n*)	Median Overall Survival (Months)	Log-Rank (Mantel–Cox) Test	Gehan–Breslow–Wilcoxon Test	Hazard Ratio (95% CI)
					χ^2^ (*p* Value)	χ^2^ (*p* Value)	
Nuclear VDR	Absent	9	8	13.3	5.60 (0.02)	4.00 (<0.05)	2.47
	Present	62	30	55.3			(0.85–7.22)
Cytoplasmic VDR	Absent	22	15	13.5	4.07 (0.04)	4.63 (0.03)	1.92
	Present	49	23	55.3			(0.53–4.00)
Either nuclear or cytoplasmic VDR	Absent	9	8	13.5	5.63 (0.02)	4.00 (<0.05)	2.47
	Present	62	30	55.3			(0.85–7.22)

HR, hazard ratio for VDR absent *vs.* VDR present; 95% CI–95% confidence interval for HR, *n*, number of patients.

Similarly, both nuclear and cytoplasmic VDR immunostaining were also statistically significant as they relate to better prognosis after adjustment for age and sex, and for age, sex, and advancement (Table 2).

**Figure 3:**
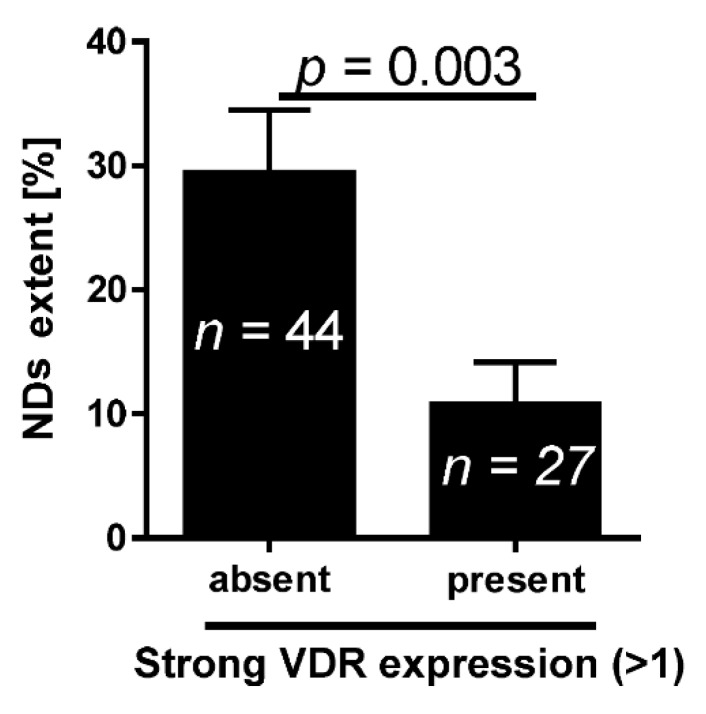
Mean non–classic differentiations extent in relation to the presence of strong cytoplasmic expression of VDR. *n*, number of patients.

We also analyzed VDR expression in relation to the non–classic differentiations (NDs, defined in Experimental Section), which reflected the capacity for multidirectional differentiation and greater risk of metastatic disease and death. A lack of high cytoplasmic VDR expression level (evaluated as 2 or 3 arbitrary units [AU]) was related to a greater extent of NDs (Figure 3).

**Table 2:** Multivariate-adjusted RRs with 95% CIs of death in relation to nuclear and cytoplasmic VDR immunostaining in urinary bladder cancers.

Adjustment	χ^2^	*p* Value	Variable	b	SE	*p* Value	Exp(b)	95% CI
**Nuclear VDR**								
Age and gender	9.45	0.024	Nuclear VDR	−0.85	0.40	0.04	0.43	0.20–0.94
			Age	0.04	0.02	0.03	1.05	1.01–1.09
			Gender *	0.10	0.43	0.81	1.10	0.48–2.56
Age, gender and advancement	17.13	0.002	Nuclear VDR	−1.10	0.41	0.01	0.34	0.15–0.75
			Age	0.04	0.02	0.04	1.05	1.00–1.09
			Gender *	0.10	0.43	0.81	1.10	0.48–2.56
			Advancement **	1.12	0.45	0.01	3.09	1.27–7.49
Age, gender and metastases	14.24	0.01	Nuclear VDR	−0.56	0.47	0.23	0.60	0.23–1.42
			Age	0.05	0.02	0.03	1.05	1.00–1.10
			Gender *	−0.27	0.49	0.58	0.76	0.30–2.00
			Metastases ***	0.98	0.37	0.01	2.67	1.29–5.51
Age, gender, advancement, the presence of metastases	17.22	0.004	Nuclear VDR	−0.79	0.50	0.11	0.45	0.17–1.17
			Age	0.05	0.02	0.04	1.05	1.00–1.10
			Gender *	−0.12	0.50	0.81	0.89	0.34–2.32
			Advancement **	0.81	0.49	0.10	2.24	0.86–5.87
			Metastasis ***	0.68	0.40	0.09	1.97	0.89–4.33
**Cytoplasmic VDR**								
Age and gender	9.95	0.02	Cytoplasmic VDR	−0.72	0.34	0.03	0.49	0.25–0.94	
			Age	0.05	0.02	0.02	1.05	1.01–1.09	
			Gender *	0.10	0.43	0.81	1.11	0.48–2.58	
Age, gender and advancement	15.98	0.003	Cytoplasmic VDR	−0.75	0.34	0.03	0.47	0.24–0.92	
			Age	0.05	0.02	0.03	1.05	1.00–1.09	
			Gender *	0.16	0.43	0.71	1.17	0.50–2.73	
			Advancement **	0.99	0.45	0.03	2.70	1.13–6.46	
Age, gender and metastases	17.13	0.002	Cytoplasmic VDR	−0.43	0.37	0.25	0.65	0.32–1.34	
			Age	0.05	0.02	0.02	1.05	1.01–1.10	
			Gender *	−0.26	0.49	0.59	0.77	0.30–2.00	
			Metastases ***	0.91	0.38	0.02	2.50	1.18–5.26	
Age, gender, advancement, the presence of metastases	16.72	0.005	Cytoplasmic VDR	−0.52	0.38	0.17	0.59	0.28–1.25	
			Age	0.05	0.02	0.04	1.05	1.00–1.10	
			Gender *	−0.11	0.50	0.82	0.89	0.34–2.36	
			Advancement **	0.72	0.48	0.13	2.05	0.81–5.22	
			Metastasis ***	0.66	0.40	0.10	1.94	0.89–4.26	

b, regression coefficient; SE, standard error; Exp(b), relative risk; 95% CI, 95% confidence interval for Exp(b); *, Gender: Male (=0) *vs.* Female (=1); **, pTa-pT2 (=0) *vs.* pT2b-pT4 (=1); ***, Metastases absent (=0) *vs.* Metastases present (=1).

**Figure 4:**
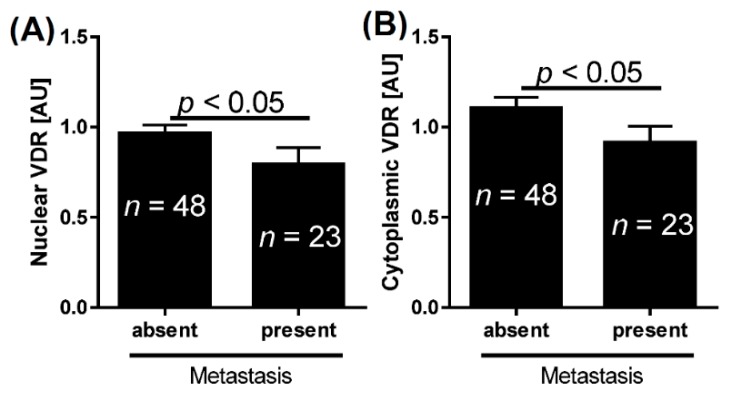
Mean nuclear (**A**) and cytoplasmic (**B**) vitamin D receptor (VDR) level in non-metastasizing and metastasizing bladder cancers. AU, arbitrary units; *n*, number of patients.

Both the nuclear and cytoplasmic VDR immunoreactivities were significantly lower in primary bladder cancers that metastasized in comparison to primary non-metastasizing tumors (Figure 4).

VDR immunostaining did not correlate with the histological tumor grade, or mitotic index.

### 2.2. CYP2B1 and Bladder Cancer

**Figure 5:**
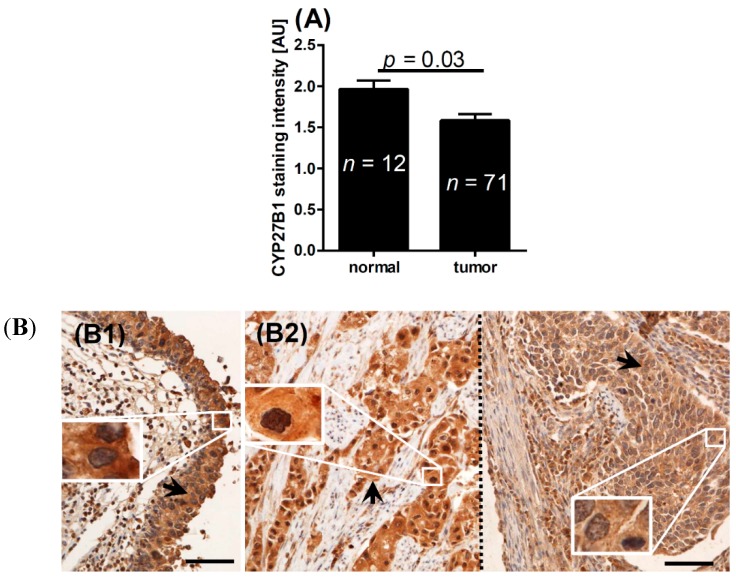
(**A**) Mean CYP27B1 level in normal epithelium and in tumors; (**B**) representative CYP27B1 staining in normal epithelium (**B1**) and cancers (**B2**). Arrows indicate CYP27B1 immunostaining, the dotted line separates photos from two different cancers (pT2a (**left**) and pT3a (**right**)). Inserts represent enlarged cells indicated by white squares. AU, arbitrary units; Scale bar: 100 μm; *n*, number of patients.

CYP27B1 expression was found both in cytoplasm of all normal epithelial cells and in 65 of 71 samples of urothelial bladder cancers (91.5%). CYP27B1 expression was significantly higher in the normal epithelium than in cancer cells (Figure 5A,B). However, CYP27B1 expression in bladder cancer did not correlate with pT stage, presence of metastasis, tumor grade, mitotic activity, and OS (not shown). In addition, there was no correlation between VDR and CYP27B1 immunostaining in bladder cancer.

The summary of the results is presented in Table 3.

**Table 3:** Summary of the results related to VDR and CYP27B1 expression in urinary bladder cancers.

Feature		Nuclear VDR		Cytoplasmic VDR		CYP27B1	
		Mean (AU)	*p* Value	Mean (AU)	*p* Value	Mean (AU)	*p* Value
Normal epithelium		1.3	<0.001 *	1.0	0.2 *	2.0	0.03 *
Normal epithelium near tumor		1.2	0.01 *	1.1	0.5 *	1.9	0.1 *
Tumor		0.9	–	1.1		1.6	–
pT	pTa-pT2a	0.8	0.3	1.4	0.005	1.6	0.06
	pT2b-pT4	0.7		1.0		1.8	
Metastasis	absent	1.0	<0.05	1.1	<0.05	1.6	0.1
	present	0.8		0.9		1.8	
Tumor grade	high	0.9	0.3	0.8	0.2	1.7	0.07
	low	0.9		1.0		1.5	
Mitosis	<10 mitoses per 1000 tumor cells	1.1	0.08	1.0	0.2	1.5	0.2
	>10 mitoses per 1000 tumor cells	0.9		0.8		1.7	

* *p* value for comparison with tumor samples.

## 3. Discussion

In the present study, we analyzed the association between the immunohistochemically-evaluated VDR and CYP27B1 levels and clinical outcomes and tumor behavior. There was a significantly longer OS for tumors with higher VDR levels in both the cytoplasm and nuclei. A higher cytoplasmic VDR level was also found in noninvasive tumors and tumors that invaded the upper layers of the muscularis propria (pTa–pT2a) compared with more advanced lesions (pT2b–pT4). The presence of a high cytoplasmic VDR level was also observed in cancers with a smaller percentage of non-classic differentiations. Both cytoplasmic and nuclear VDR staining was significantly higher in bladder cancers that did not develop metastasis compared with those that metastasized. No relationship between VDR expression and histological markers of malignancy such as a histological grade and mitotic activity was observed. The reduced expression of CYP27B1 was found in tumor cells, but its expression did not correlate with pT stage, presence of metastasis, tumor grade, mitotic activity or OS.

Previously we have observed a positive association between low VDR expression and increasing tumor stage in cutaneous melanomas (Breslow thickness, Clark level, pTNM stage, overall stage, and presence of negative prognostic markers) with lack of or low VDR expression determining poorer prognosis [19,32]. A similar relationship was found for CYP27B1 expression in melanomas and ovarian cancers [18,33]. Although in the present study we did not find a linear correlation between VDR level and increasing pT advancement, the differences in VDR levels between bladder cancers subgrouped as pTa–pT2a and pT2b–pT4 evidenced negative correlation between tumor progression and VDR expression. Therefore, we suggest that crossing of the deeper layers of the muscularis propria by bladder cancer cells and acquisition of metastatic potential are linked to a loss or significant reduction of VDR expression. This hypothesis is supported by the observation of lower VDR levels in metastasizing bladder cancers.

VDR belongs to a superfamily of nuclear receptors. However, in previous [19,32] and present studies we observed both nuclear and cytoplasmic VDR immunostaining. Correspondingly, in other non-malignant and malignant tissues similar VDR immunolocalization was found [33,34,35]. Such localization is consistent with function of VDR, which after binding 1,25(OH)_2_D3 in the cytoplasm, heterodimerizes with retinoic acid X receptor and is translocated to the nucleus [7].

In the tested bladder cancer samples, VDR expression was not related to several defined histological markers of malignancy such as mitotic activity or histological grade, whereas the presence of VDR in bladder cancer cells was related to longer OS. This suggests that the association between the expression of the VDR and bladder tumor biology is complex. Since vitamin D is a positive regulator of VDR expression [36,37], we cannot exclude the possibility that the VDR levels in bladder cancer cells are related to and/or regulated by the local and systemic vitamin D levels. Thus, the lower VDR expression could reflect vitamin D insufficiency, which led to poor outcomes in these bladder cancer patients. This concept seems to be supported by recent reports showing that vitamin D status determines surgical outcomes, and suggest that vitamin D supplementation may help to improve patient safety at the time of surgery (e.g., by reducing in-hospital mortality/morbidity, serious infections, and serious cardiovascular events) as it was observed after transplantations, cardiac, non-cardiac, orthopedic surgeries, and other procedures [38,39]). However, lack of correlation between CYP27B1 expression, a required step of vitamin D activation, and tumor progression and OS raises additional possibilities. Recently new alternative pathways of vitamin D3 activation by CYP11A1 were discovered producing vitamin D3 hydroxyderivatives [40,41,42,43] that do not require C1α-hydroxylation for their biological activity [40,44,45]. Thus, the significant effects of VDR expression on survival and metastatic potential observed in our study could represent a combined activity of several active vitamin D metabolites independent of CYP27B1, in addition to 1,25(OH)_2_D_3_, which requires CYP27B1 activity.

One of the first report related to VDR immunostaining in urinary bladder cancers was published by Sahin *et al.* [46]. However authors analyzed only superficial tumors. In our study we included both superficial and invasive tumors. Additionally, we analyzed VDR immunostaining in relation to prognostic factors such as tumor advancement, mitotic activity and most importantly we related the findings to the overall survival time. We also applied different methods of VDR immunostaining assessment.

Zhou *et al.* [47] observed diverse expression of VDR in esophageal adenocarcinomas and squamous cell cancers, with higher expression in the former. In the adenocarcinomas authors found no relationship between VDR expression and tumor stage, metastases and survival. However, it could not be excluded that these differences in VDR protein level were affected by histological origin of cancer cells. In urothelial bladder cancers, the capacity for multidirectional differentiation as determined by the NDs is accompanied by greater tumor malignancy and the ability of a tumor to metastasize [48,49,50]. In the present study, a significant reduction in VDR level was found in cancers with wider extension of NDs. This correlation suggests that disturbances of VDR expression might affect bladder cancer cell differentiation and that a lack of or decrease in VDR expression may make these cells unresponsive to the antitumorigenic action of active forms of vitamin D.

Population-based studies revealed an importance of VDR gene polymorphism in cancer biology and that selected VDR polymorphism altered susceptibility and prognosis of different tumors [51]. The studies of VDR polymorphism in urinary bladder cancers are sparse. Very recently published report showed that rs731236 variant of the VDR showed a protective effect for male lower urinary tract symptoms [52]. Increased risk for bladder cancers seems to be associated with Fok-I VDR polymorphism [53]. Although we did not study VDR gene polymorphism, our study on correlation between VDR protein expression and tumor behavior are consistent with the above reports and together indicate a protective role for proper VDR signaling against oncogenesis or tumor progression. Renal dysfunction, including insufficient glomerular filtration rate is the condition excluding the possibility of adjuvant cisplatin-based combination chemotherapy of urinary bladder cancer patients [5,6]. Renal diseases can affect vitamin D activation, since the second step of vitamin D hydroxylation catalyzed by CYP27B1 takes place in kidney cells [7] and could decrease the usefulness vitamin D supplementation in cancer patients. However a number of other tissues also express the CYP27B1 enzyme, including bladder and skin cells [54], which could compensate the insufficient activation of 25(OH)D3 due to renal impairment.

Additionally, some studies indicate that smooth muscle cells of urinary bladders are sensitive to the action of VDR agonist regulating its anti-inflammatory properties [55,56]. Thus, vitamin D-based treatment could have broader effects, which are not solely limited to treatment of urinary bladder cancer. The effects of vitamin D on bladder cancer can be determined by biological properties of cancer cells as was observed by Ma *et al*. [57], and mediated by regulation of miRNA, differentially-regulated by vitamin D in metastatic and nonmetastatic bladder cancer cells [57].

Our studies represent the retrospective immunostaining analyses in routine, formalin-fixed paraffin-embedded material from a relatively small patient cohort with urinary bladder cancer (71 patients). Therefore, further validations on independent and larger population of patients with urinary bladder cancer are needed. However, our analysis of VDR and CYP27B1 immunostaining in urinary bladder cancers is very comprehensive and clinically significant, because of inclusion of data on overall survival time, which also correlated with histological parameters. Into this study we qualified patients with both superficial and invasive tumors, and the basic criterion of qualification was the availability of material (cystectomy, histologically diagnosed tumor, representative tumor mass in the tissue section). No other clinical or pathomorphological features were considered at this step. In the future we plan to perform prospective study on vitamin D serum level, VDR polymorphisms and epigenetic regulation of VDR and CYP27B1 expression in in patients with bladder cancer.

Considering our results and published data, we propose that vitamin D may act as an adjuvant in bladder cancer treatment. This hypothesis is consistent with the studies by Zeichner *et al.* [31], who observed improved DFS in HER-positive non-metastatic breast cancer patients who received vitamin D supplementation. Thus routine anticancer therapy with systemic application of vitamin D3 or transurethral application of active forms of vitamin D3 could be an efficient mode of action resulting in the longer survival of VDR-positive bladder cancer patients.

## 4. Experimental Section

### 4.1. Patients and Pathological Morphological Assessment

The study was approved by the Committee of Ethics of Scientific Research of Collegium Medicum of Nicolaus Copernicus University in Toruń, Poland (approval number KB 446/2009, September 2009). All patients who underwent cystectomy or cystoprostatectomy in the Oncology Centre–Prof. Franciszek Łukaszczyk Memorial Hospital, Bydgoszcz, Poland, from 2007 to 2010 were included into this study. In the next step of qualification, patients without tumor in resected urinary bladder or with insufficient tumor presence in blocks after diagnostic procedures were excluded. Finally seventy-one patients (mean age 64.7 years, range 47.2–83.8 years) were included in this study. The characteristic of the patients recruited to this study are presented in Table 4. The clinical–pathomorphological data were obtained from the electronic database of the Oncology Center, Bydgoszcz, Poland. The dates of deaths were obtained from the Department of Registry Office in Bydgoszcz, Poland, and from the Polish National Cancer Registry. Information concerning the age, gender, date of diagnosis, survival or the date of death were available for each patient included into this study. The survival time was calculated based on date of pathomorphological diagnosis of bladder cancer and the date of death or the end of observation. The patients who did not die were censored. The mean follow up time was 46.7 months, and the mean survival time was 35.5 months.

Pathological assessment (p) of primary tumors advancement (T) was performed according to World Health Organization (WHO) Classification of Malignant Tumors [1]: pTa: papillary tumor extent confined to urothelium, pTis: non-papillary tumor extent confined to urothelium, pT1: tumor extent confined to mucosa, pT2a: tumor invades superficial muscularis propria (inner half), pT2b: tumor invades deep *muscularis propria* (outer half), pT3: tumor invades perivesical fat, and pT4: tumor invades perivesical organs. Histological maturity (grade) was evaluated according to WHO TNM Classification of Malignant Tumors [1] as follows: low grade of histological maturity was classified as high-grade tumors and high level of histological differentiation as low-grade tumors. Mitotic activity was assessed by counting mitosis in tumor cells per 1000 tumor cells.

The presence of pathomorphologically confirmed tumors other than bladder cancer (colorectal and prostate cancers and lymphoma) was also recorded.

From each patients’ resected urinary bladder tissue the tissue block with representative tumor presentation was selected. In addition, normal samples (*n* = 12), with representative normal epithelium, that were localized more than 2 cm from the tumor and collected separately from tumor samples during routine histological procedures, were included. Nine of 12 normal samples were paired with tumor samples included in these analyses. Furthermore, normal epithelial cells that were present in the same section as tumor cells and were localized 0.5–2 cm from the tumor were defined as normal epithelium near tumor and analyzed in relation to VDR immunostaining (*n* = 29).

The qualification of cases as metastatic or non-metastatic was based on pathomorphological assessment of tumor cells presence in lymph nodes or other organs.

**Table 4:** Clinical-pathomorphological characteristics of patients with urothelial bladder cancer.

Feature		Number of Patients
**Gender**	female	13
	males	58
**pT** ^a^	a	5
	is	1
	1	12
	2a	3
	2b	14
	3	24
	4	12
**NDs** ^b^	Absent	32
	Present	39
**Grade**	Low grade	19
	High grade	52
**Metastases**	Absent	44
	Present	27
**Second tumor**	Absent	62
	Present	9
**Concomitant carcinoma *in situ***	Absent	64
	Present	7
**Survival**	Alive	33
	Death	38

^a^ pT, pathomorphological assessment of primary tumor; ^b^ NDs, non–classic differentiations.

### 4.2. Immunohistochemical Staining and Evaluation of the VDR

The VDR was detected using immunohistochemistry as previously described [19,32]. Briefly, standard formalin-fixed, paraffin-embedded 4 μm sections were incubated overnight with rat anti–VDR antibody (clone 97A; Abcam Inc., Cambridge, MA, USA), after which the sections were stained with anti–rat secondary antibody conjugated with alkaline phosphatase (Vector Laboratories Inc., Burlingame, CA, USA). The sections were then incubated with red alkaline phosphatase substrate and mounted in an aqueous medium (Dako, Glostrup, Denmark).

CYP27B1 was detected as previously described [18,33]. Briefly, rabbit anti–CYP27B1 antibody (Santa Cruz Biotechnology, Santa Cruz, CA, USA) at a dilution of 1:75 was applied, and the samples were incubated overnight at 4 °C with anti–CYP27B1 antibody. This was followed by staining with horseradish peroxidase (HRP)-labeled anti-rabbit antibody, 3,3′-diaminobenzidine (Envision System HRP-Labeled Polymer Anti–Mouse; Dako, Glostrup, Denmark), and hematoxylin counterstaining.

**Figure 6:**
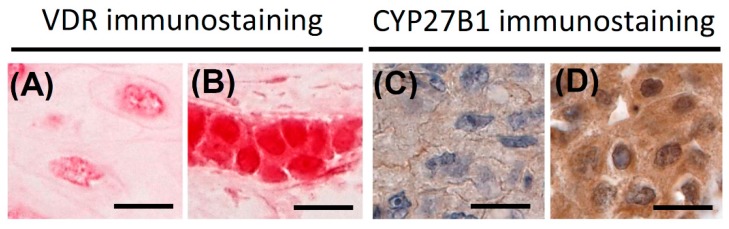
Representative immunostaining of VDR and CYP27V1. (**A**) Cytoplasmic VDR assessed as zero, nuclear VDR as one; (**B**) cytoplasmic VDR assessed as two, nuclear VDR as three; (**C**) CYP27B1 assessed as zero; and (**D**) CYP27B1 assessed as three. Scale bar: 200 µm.

Evaluation of immunostained sections was performed by two independent observers (Wojciech Jóźwicki and Anna A. Brożyna) in a blind manner without knowing the histopathological diagnosis, pathomorphological features, and other clinical data. The cytoplasmic and nuclear VDR and cytoplasmic CYP27B1 immunostaining intensity was scored semi-quantitatively (considering both staining intensity and percentage of cells using a four-point scale of zero to three arbitrary units (AU) as previously described [32,33]). Briefly, VDR staining intensity was evaluated with reference to intense reddish pink basal layer of normal skin epidermis, served as control, scored as strong (staining intensity three). Light reddish pink and light pink stained cells were scored as cells with moderate (staining intensity two) or weak (staining intensity one) VDR expression, correspondingly. The lack of staining was assessed as negative (staining intensity zero) (Figure 6A,B). CYP27B1 staining intensity was scored semi-quantitatively with zero as negative (zero), weak (one), moderate (two) and strong (three) (Figure 6C,D). Staining intensity was evaluated with reference to immunostaining of normal skin epidermis, scored as strong, served as control. The semi-quantitative score were calculated according the following formula: SQ = mean(IR × SI)/100, where IR is the percentage of immunoreactive cells and SI is the staining intensity.

Patients were stratified according to semi-quantitative score as follows: absent VDR or CYP27B1 ≤ 0.99, present VDR or CYP27B1 > 0.99.

Since in previous study [19,32] we found immunohistochemical localization of VDR both in cell nuclei and cytoplasm, in this study we also assessed nuclear and cytoplasmic VDR staining in normal and tumor epithelial cells. We evaluated cytoplasmic and nuclear VDR staining separately. Additionally, combined data representing either nuclear or cytoplasmic VDR expression was used for the analysis of overall survival time. VDR and CYP27B1 was analyzed with a continuous measure of staining intensity and dichotomized by “absent” or “present”, as defined above.

VDR and CYP27B1 staining was analyzed in relation to the presence of prognostic markers such as tumor advancement, presence of metastases, tumor grade, mitotic activity, and clinical outcome evaluated as survival time.

### 4.3. Evaluation of the NDs

In routine hematoxylin and eosin-stained sections, the presence of a non-classic differentiations (NDs) was identified according to the histological classification of urinary tumors of the World Health Organization [1]. The percentage tissue displaying the non–classic differentiation pattern, which was defined as the average percentage of the surface of the whole neoplastic tissue displaying that pattern, was recorded for the sections of the tumors examined [46].

### 4.4. Statistical Analyses

Statistical analysis was performed using Prism 6.05 (GraphPad Software Inc, San Diego, CA, USA and MedCalc Version 15.8 (MedCalc Software bvba, Ostend, Belgium). The results were considered as significant at *p* < 0.05. The data are presented as mean ± SD. For comparison of two variables, the Mann–Whitney *U* test was used. Survival time was analyzed using Kaplan–Meier curves. Overall survival was also compared using the log-rank and Gehan–Wilcoxon tests. In addition, Cox proportional hazards regression was used to compare the mortality rates between nuclear and cytoplasmic VDR-positive and negative patients, and calculated as a relative risk (Exp(b)). Relative risks were adjusted for age, gender, tumor advancement (pTa-pT2a *vs.* pT2b-pT4 tumors), and the presence of metastases.

## 5. Conclusions

In summary, VDR and CYP27B1 expression was down-regulated in urothelial bladder cancers in comparison to normal tissue. There was a strong relationship between VDR expression and the biology and stage of bladder cancers, with a lack of such correlation for CYP27B1. Urothelial bladder cancer progression and decrease of overall survival were linked with a reduction in VDR expression. These results have potential clinical implications in that a decreased VDR expression may predict a poorer prognosis, as demonstrated by the lower overall survival in our study. VDR expression can also be a potential prognostic marker in urothelial bladder cancer patients. The lack of correlation between VDR expression and some histological markers of malignancy such as mitotic activity and tumor grade and lack of correlation with CYP27B1 indicate a more complex role of the VDR signaling in the biology of bladder cancer that may include involvement of the new vitamin metabolites activating alternative pathways. The differences in VDR expression in bladder cancer suggest that vitamin D-based therapies may represent a promising previously unexplored strategies of bladder cancer treatment.

## References

[1] Lopez-Beltran A., Sauter G., Gasser T., Hartmann A., Schmitz-Dräger B.J., Helpap B., Ayala A.G., Tamboli P., Knowles M.A., Sidransky D., Eble J.N., Sauter G., Epstein J.I., Sesterhenn I.A. (2004). Tumours of the urinary system. Infiltrating urothelial carcinoma. WHO Classification of Tumours. Pathology and Genetics. Pathology and Genetics of Tumors of the Urinary System and Male Genital Organs.

[2] Domanowska E., Jozwicki W., Domaniewski J., Golda R., Skok Z., Wisniewska H., Sujkowska R., Wolski Z., Jozwicka G. (2007). Muscle-invasive urothelial cell carcinoma of the human bladder: Multidirectional differentiation and ability to metastasize. Hum. Pathol..

[3] Siegel R., Ma J., Zou Z., Jemal A. (2014). Cancer statistics, 2014. CA Cancer J. Clin..

[4] Madersbacher S., Hochreiter W., Burkhard F., Thalmann G.N., Danuser H., Markwalder R., Studer U.E. (2003). Radical cystectomy for bladder cancer today—A homogeneous series without neoadjuvant therapy. J. Clin. Oncol..

[5] Niegisch G., Lorch A., Droller M.J., Lavery H.J., Stensland K.D., Albers P. (2012). Neoadjuvant chemotherapy in patients with muscle-invasive bladder cancer: Which patients benefit?. Eur. Urol..

[6] Thompson R.H., Boorjian S.A., Kim S.P., Cheville J.C., Thapa P., Tarrel R., Dronca R., Costello B., Frank I. (2014). Eligibility for neoadjuvant/adjuvant cisplatin-based chemotherapy among radical cystectomy patients. BJU Int..

[7] Holick M.F. (2007). Vitamin D Deficiency. N. Engl. J. Med..

[8] Gröschel C., Tennakoon S., Kállay E. (2015). Cytochrome P450 vitamin D hydroxylases in inflammation and cancer. Adv. Pharmacol..

[9] Hossein-nezhad A., Holick M.F. (2013). Vitamin D for health: A global perspective. Mayo Clin. Proc..

[10] Cieslak K., Feingold J., Antonius D., Walsh-Messinger J., Dracxler R., Rosedale M., Aujero N., Keefe D., Goetz D., Goetz R. (2014). Low vitamin D levels predict clinical features of schizophrenia. Schizophr. Res..

[11] Koplin J.J., Suaini N.H., Vuillermin P., Ellis J.A., Panjari M., Ponsonby A.L., Peters R.L., Matheson M.C., Martino D., Dang T. (2015). Polymorphisms affecting vitamin D-binding protein modify the relationship between serum vitamin D (25[OH]D3) and food allergy. J. Allergy. Clin. Immunol..

[12] Rose K., Penna-Martinez M., Klahold E., Kärger D., Shoghi F., Kahles H., Bayer M., Hintermann E., Pfeilschifter J.M., Badenhoop K. (2013). Influence of the vitamin D plasma level and vitamin D-related genetic polymorphisms on the immune status of patients with type 1 diabetes: A pilot study. Clin. Exp. Immunol..

[13] Jeon K., Kim S.Y., Jeong B.H., Chang B., Shin S.J., Koh W.J. (2013). Severe vitamin D deficiency is associated with non-tuberculous mycobacterial lung disease: A case-control study. Respirology.

[14] Maalmi H., Ordóñez-Mena J.M., Schöttker B., Brenner H. (2014). Serum 25-hydroxyvitamin D levels and survival in colorectal and breast cancer patients: Systematic review and meta-analysis of prospective cohort studies. Eur. J. Cancer.

[15] Oh J.J., Byun S.S., Lee S.E., Hong S.K., Jeong C.W., Choi W.S., Kim D., Kim H.J., Myung S.C. (2014). Genetic variants in the CYP24A1 gene are associated with prostate cancer risk and aggressiveness in a Korean study population. Prostate Cancer Prostatic Dis..

[16] Agic A., Xu H., Altgassen C., Noack F., Wolfler M.M., Diedrich K., Friedrich M., Taylor R.N., Hornung D. (2007). Relative expression of 1,25-dihydroxyvitamin D3 receptor, vitamin D 1α-hydroxylase, vitamin D 24-hydroxylase, and vitamin D 25-hydroxylase in endometriosis and gynecologic cancers. Reprod. Sci..

[17] Brożyna A.A., Jochymski C., Janjetovic Z., Jozwicki W., Tuckey R.C., Slominski A.T. (2014). CYP24A1 expression inversely correlates with melanoma progression: Clinic-pathological studies. Int. J. Mol. Sci..

[18] Brożyna A.A., Jozwicki W., Jochymski C., Slominski A.T. (2015). Decreased expression of CYP27B1 correlates with the increased aggressiveness of ovarian carcinomas. Oncol. Rep..

[19] Brożyna A.A., Jozwicki W., Slominski A.T. (2014). Decreased VDR expression in cutaneous melanomas as marker of tumor progression: New data and analyses. Anticancer Res..

[20] Clinckspoor I., Verlinden L., Overbergh L., Korch C., Bouillon R., Mathieu C., Verstuyf A., Decallonne B. (2012). 1,25-dihydroxyvitamin D3 and a superagonistic analog in combination with paclitaxel or suberoylanilide hydroxamic acid have potent antiproliferative effects on anaplastic thyroid cancer. J. Steroid Biochem. Mol. Biol..

[21] Amaral A.F., Mendez-Pertuz M., Munoz A., Silverman D.T., Allory Y., Kogevinas M., Lloreta J., Rothman N., Carrato A., Rivas del Fresno M. (2012). Plasma 25-hydroxyvitamin D3 and bladder cancer risk according to tumor stage and FGFR3 status: A mechanism-based epidemiological study. J. Natl. Cancer Inst..

[22] Chen F., Li Q., Yu Y., Yang W., Shi F., Qu Y. (2015). Association of vitamin C, vitamin D, vitamin E and risk of bladder cancer: A dose–response meta-analysis. Sci. Rep..

[23] Liao Y., Huang J.L., Qiu M.X., Ma Z.W. (2015). Impact of serum vitamin D level on risk of bladder cancer: A systemic review and meta-analysis. Tumour Biol..

[24] Mondul A.M., Weinstein S.J., Mannisto S., Snyder K., Horst R.L., Virtamo J., Albanes D. (2010). Serum vitamin D and risk of bladder cancer. Cancer Res..

[25] Janjetovic Z., Tuckey R.C., Nguyen M.N., Thorpe E.M., Slominski A.T. (2010). 20,23-dihydroxyvitamin D3, novel p450scc product, stimulates differentiation and inhibits proliferation and NF-κB activity in human keratinocytes. J. Cell. Physiol..

[26] Slominski A.T., Janjetovic Z., Kim T.K., Wright A.C., Grese L.N., Riney S.J., Nguyen M.N., Tuckey R.C. (2012). Novel vitamin D hydroxyderivatives inhibit melanoma growth and show differential effects on normal melanocytes. Anticancer Res..

[27] Tieu E.W., Tang E.K., Chen J., Li W., Nguyen M.N., Janjetovic Z., Slominski A., Tuckey R.C. (2012). Rat CYP24A1 acts on 20-hydroxyvitamin D_3_ producing hydroxylated products with increased biological activity. Biochem. Pharmacol..

[28] Wang J., Slominski A., Tuckey R.C., Janjetovic Z., Kulkarni A., Chen J., Postlethwaite A.E., Miller D., Li W. (2012). 20-Hydroxyvitamin D3 inhibits proliferation of cancer cells with high efficacy while being non-toxic. Anticancer Res..

[29] Alibhai S.M., Mohamedali H.Z., Gulamhusein H., Panju A.H., Breunis H., Timilshina N., Fleshner N., Krahn M.D., Naglie G., Tannock I.F. (2013). Changes in bone mineral density in men starting androgen deprivation therapy and the protective role of vitamin D. Osteoporos. Int..

[30] Dueregger A., Heidegger I., Ofer P., Perktold B., Ramoner R., Klocker H., Eder I.E. (2015). The use of dietary supplements to alleviate androgen deprivation therapy side effects during prostate cancer treatment. Nutrients.

[31] Zeichner S.B., Koru-Sengul T., Shah N., Liu Q., Markward N.J., Montero A.J., Gluck S., Silva O., Ahn E.R. (2015). Improved clinical outcomes associated with vitamin D supplementation during adjuvant chemotherapy in patients with HER2^+^ nonmetastatic breast cancer. Clin. Breast Cancer.

[32] Brożyna A.A., Jozwicki W., Janjetovic Z., Slominski A.T. (2011). Expression of vitamin D receptor decreases during progression of pigmented skin lesions. Hum. Pathol..

[33] Brożyna A.A., Jozwicki W., Janjetovic Z., Slominski A.T. (2013). Expression of the vitamin D-activating enzyme 1α-hydroxylase (CYP27B1) decreases during melanoma progression. Hum. Pathol..

[34] Tokumoto M., Tsuruya K., Fukuda K., Kanai H., Kuroki S., Hirakata H. (2002). Reduced p21, p27 and vitamin D receptor in the nodular hyperplasia in patients with advanced secondary hyperparathyroidism. Kidney Int..

[35] Menezes R.J., Cheney R.T., Husain A., Tretiakova M., Loewen G., Johnson C.S., Jayaprakash V., Moysich K.B., Salgia R., Reid M.E. (2008). Vitamin D receptor expression in normal, premalignant, and malignant human lung tissue. Cancer Epidemiol. Biomark. Prev..

[36] Pike J.W., Meyer M.B. (2010). The vitamin D receptor: New paradigms for the regulation of gene expression by 1,25-dihydroxyvitamin D_3_. Endocrinol. Dis. Clin. N. Am..

[37] Pike J.W., Lee S.M., Meyer M.B. (2014). Regulation of gene expression by 1,25-dihydroxyvitamin D3 in bone cells: exploiting new approaches and defining new mechanisms. BoneKEy Rep..

[38] Iglar P.J., Hogan K.J. (2015). Vitamin D status and surgical outcomes: A systematic review. Patient Saf. Surg..

[39] Turan A., Hesler B.D., You J., Saager L., Grady M., Komatsu R., Kurz A., Sessler D.I. (2014). The association of serum vitamin D concentration with serious complications after noncardiac surgery. Anesth. Analg..

[40] Slominski A., Kim T.K., Li W., Yi A.K, Postlethwaite A., Tuckey R.C. (2014). The physiological role of CYP11A1 in vitamin D regulation of epidermal functions. J. Steroid Biochem. Mol. Biol..

[41] Slominski A.T., Kim T.-K., Shehabi H.Z., Semak I., Tang E.K., Nguyen M.N., Benson H.A., Korik E., Janjetovic Z., Chen J. (2012). *In vivo* evidence for a novel pathway of vitamin D3 metabolism initiated by P450scc and modified by CYP27B1. FASEB J..

[42] Slominski A.T., Li W., Kim T., Semak I., Wang J., Zjawiony J., Tuckey R.C. (2015). Novel activities of CYP11A1 and their potential physiological significance. J. Steroid Biochem. Mol. Biol..

[43] Slominski A.T., Janjetovic Z., Kim T.-K., Wasilewski P., Rosas S., Hanna S., Sayre R.M., Dowdy J.C., Li W., Tuckey R.C. (2015). Novel non-calcemic secosteroids that are produced by human epidermal keratinocytes protect against solar radiation. J. Steroid Biochem. Mol. Biol..

[44] Slominski A.T., Kim T.-K., Janjetovic Z., Tuckey R.C., Bieniek R., Yue Y., Li W., Chen J., Miller D., Chen T. (2011). 20-Hydroxyvitamin D2 is a non-calcemic analog of vitamin D with potent antiproliferative and prodifferentiation activities in normal and malignant cells. Am. J. Physiol. Cell Physiol..

[45] Slominski A., Kim T.-K., Takeda Y., Janjetovic Z., Brożyna A.A., Skobowiat C., Wang J., Postlethwite A., Li W., Tuckey R.C. (2014). RORα and RORγ are expressed in human skin and serve as receptors for endogenously produced noncalcemic 20-hydroxy- and 20,23-dihydroxy-vitamin D. FASEB J..

[46] Sahin M.O., Canda A.E., Yorukoglu K., Mungan M.U., Sade M., Kirkali Z. (2005). 1,25 Dihydroxyvitamin D_3_ receptor expression in superficial transitional cell carcinoma of the bladder: A possible prognostic factor?. Eur. Urol..

[47] Zhou Z., Xia Y., Bandla S., Zakharov V., Wu S., Peters J., Godfrey T.E., Sun J. (2014). Vitamin D receptor is highly expressed in precancerous lesions and esophageal adenocarcinoma with significant sex difference. Hum. Pathol..

[48] Jozwicki W., Brożyna A.A., Siekiera J. (2014). Expression of OCT4a: The first step to the next stage of urothelial bladder cancer progression. Int. J. Mol. Sci..

[49] Jozwicki W., Brożyna A.A., Siekiera J., Slominski A.T. (2015). Expression of RCAS1 correlates with urothelial bladder cancer malignancy. Int. J. Mol. Sci..

[50] Jóźwicki W., Skok Z., Brożyna A., Siekiera J., Wolski Z., Domaniewski J. (2010). Prognostic and diagnostic implications of histological differentiation in invasive urothelial cell carcinoma of the bladder: Variant or non-classic differentiation number. Cent. Eur. J. Urol..

[51] Gandini S., Gnagnarella P., Serrano D., Pasquali E., Raimondi S. (2014). Vitamin D receptor polymorphisms and cancer. Adv. Exp. Med. Biol..

[52] Cartwright R., Mangera A., Tikkinen K.A., Rajan P., Pesonen J., Kirby A.C., Thiagamoorthy G., Ambrose C., Gonzalez-Maffe J., Bennett P.R. (2014). Systematic review and meta-analysis of candidate gene association studies of lower urinary tract symptoms in men. Eur. Urol..

[53] Mittal R.D., Manchanda P.K., Bhat S., Bid H.K. (2007). Association of vitamin-D receptor (*Fok-I*) gene polymorphism with bladder cancer in an Indian population. BJU Int..

[54] Bikle D.D. (2014). Vitamin D metabolism, mechanism of action, and clinical applications. Chem. Biol..

[55] Crescioli C., Morelli A., Adorini L., Ferruzzi P., Luconi M., Vannelli G.B., Marini M., Gelmini S., Fibbi B., Donati S. (2005). Human bladder as a novel target for vitamin D receptor ligands. J. Clin. Endocrinol. Metab..

[56] Adorini L., Penna G., Amuchastegui S., Cossetti C., Aquilano F., Mariani R., Fibbi B., Morelli A., Uskokovic M., Colli E. (2007). Inhibition of prostate growth and inflammation by the vitamin D receptor agonist BXL-628 (elocalcitol). J. Steroid Biochem. Mol. Biol..

[57] Ma Y., Hu Q., Luo W., Pratt R.N., Glenn S.T., Liu S., Trump D.L., Johnson C.S. (2015). 1α,25(OH)_2_D_3_ differentially regulates miRNA expression in human bladder cancer cells. J. Steroid Biochem. Mol. Biol..

